# *Grewia asiatica* L., a Food Plant with Multiple Uses

**DOI:** 10.3390/molecules18032663

**Published:** 2013-02-28

**Authors:** Muhammad Zia-Ul-Haq, Milan S. Stanković, Komal Rizwan, Vincenzo De Feo

**Affiliations:** 1 Department of Pharmacognosy, University of Karachi, Karachi-75270, Pakistan; E-Mail: ahirzia@gmail.com; 2 Department of Biology and Ecology, Faculty of Science, University of Kragujevac, Street Radoja Domanovića No. 12, Kragujevac 34000, Serbia; E-Mail: mstankovic@kg.ac.rs; 3 Department of Chemistry, Government College University Faisalabad, Faisalabad-38000, Pakistan; E-Mail: komal.rizwan45@yahoo.com; 4 Department of Pharmacy, University of Salerno, Fisciano, Salerno 84084, Italy

**Keywords:** *Grewia asiatica*, phytochemistry, nutrition, pharmacological properties

## Abstract

*Grewia asiatica* L., is a species native to south Asia from Pakistan, east to Cambodia, cultivated primarily for its edible fruit and well-reputed for its diverse medicinal uses. Fruits are a rich source of nutrients such as proteins, amino acids, vitamins, and minerals and contain various bioactive compounds, like anthocyanins, tannins, phenolics and flavonoids. Different parts of this plant possess different pharmacological properties. Leaves have antimicrobial, anticancer, antiplatelet and antiemetic activities; fruit possess anticancer, antioxidant, radioprotective and antihyperglycemic properties; while stem bark possesses analgesic and anti-inflammatory activities. This review focuses on the botanical description, phytochemistry, nutritional studies and pharmacological properties of this plant.

## 1. Introduction

The dieto-therapeutic importance of fruits is well-recognized since the beginning of human civilization. Fruits occupy an important position in the socio-cultural and health systems of many countries and there is a growing interest in exploring their therapeutic and nutritional properties. Antioxidants present in fruits and juices made from them, are claimed to be helpful against cancer, cardiovascular and various chronic diseases. The presence of various biofunctional and chemo-preventive compounds in fruits, believed to have health-boosting properties, are a major reason for their increased consumption. Fruits are regarded as a valuable food commodity with potential health benefits, being a rich source of carbohydrates, vitamins, antioxidants and minerals which are essential for an active and healthy life.

*Grewia* genus (*Tiliaceae*) comprises approximately 150 species of small trees and shrubs, distributed in subtropical and tropical regions of World and is the only genus in the family that yields edible fruits. In Pakistan, 10 species belonging to this genus have been identified, namely *G. asiatica* L., *G. damine* Gaertn., *G. elastica* Royle, *G. glabra* Blume, *G. helicterifolia* Wall., *Grewia microcos* L., *G. optiva* J. R. Drumm. ex Burret, *G. sapida* Roxb., *G. tenax* (Forssk.) Fiori and *G. villosa* Willd [1,2,3]. *Grewia asiatica* L., grows wildly, and is also cultivated in south Asian countries. The name *Grewia* was given due to Nehemiah Grew, one of the founders of plant physiology, while *asiatica* reflects the Asian origin of this species. *G. asiatica*, locally known as *phalsa*, is well-known for its nutritional and therapeutic attributes. Despite its diverse use, it has suffered notable disregard, as is evident from the lack of literature on this plant. As a step in this direction and as part of our studies [4,5,6,7,8,9,10,11] on documenting biological and chemical studies of indigenous flora of Pakistan, we have reviewed the phytochemistry, nutritional importance and pharmacological properties of this plant. This review will serve as a useful reference for further research on this important plant.

## 2. Botanical Description and Traditional Uses

**Figure 1:**
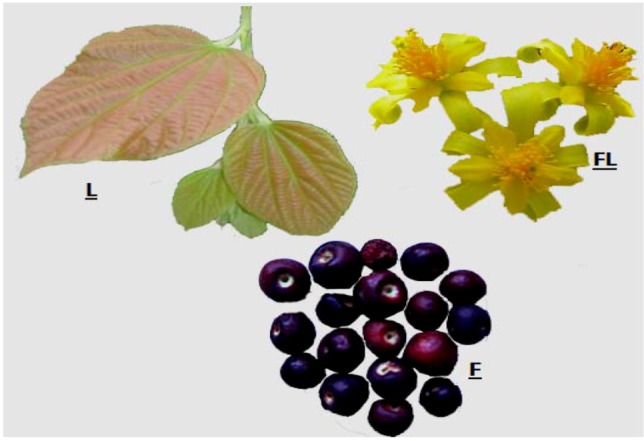
*Grewia asiatica* L., FL, flower; L, leaf, S, seed.

*Grewia asiatica* is a 4 to 5 m tall shrub. The leaves are approximately 5–18 cm long and broad. The flowers are arranged in cymes of several together, the individual flowers are yellowish in color with five large (12 mm) sepals and five smaller (4–5 mm) petals. The flower has a diameter of about 2 cm (Figure 1) [12].

Leaves may be ovate, suborbicular, acute, subacuminate or cuspidate, sharply and often coarsely, double serrate, subglabrous above, hoary-tomentose beneath and rounded or only slightly cordate at the base. Flower buds are broadly cylindric or clavate, peduncles are axillary, usually many, and long and slender. Bracts are present beneath the pedicles. The fruit is globose, 1.0 to 1.9 cm in diameter, 0.8 to 1.6 cm in vertical height, and 0.5 to 2.2 g in weight and is edible portion of plant. Plant flowers appear in January-February and fruits mature in May-June. During ripening, the fruit skin turns from light green to cherry red or purplish red, becoming dark purple or nearly black when fully ripened. The ripe fruit is soft and delicate and is covered with a very thin whitish blush [13,14,15]. The fruit is like a berry and has a sweet and sour acidic taste. The flavor is like grapes. Phalsa fruits fetch a very good price of $1/Kg in local markets and are consumed fresh with some salt, which is added to increase palatability. It is also used in making jams, pies, squashes and chutneys. However, it has a short shelf life and is considered suitable only for local marketing [16,17]. The fruits are claimed to be beneficial for heart, blood and liver disorders, anorexia, indigestion, thirst, toxemia, stomatitis, hiccough, asthma, spermatorrhoea, fevers and diarrhea and are used for treating throat, tuberculosis and sexual debility troubles [18,19,20,21,22]. The root bark is used for the treatment of rheumatism and urinary tract problems [23,24], while the stem bark is used in sugar refining [25]. The leaves are applied to the skin for wounds and cuts and to relieve irritation and painful rashes [26]. They are thought to possess an antibiotic effectd and are also used as cattle fodder. The stems and the bark can be used to make ropes, baskets and are harvested for fuel. The bark is used as a soap substitute [27].

Two distinct types, tall and dwarf, have been developed in India that differ with respect to various chemical and physical characteristics (Table 1). The juice yield is slightly higher in the tall type because it is directly related to edible portion, while more total sugars and non-reducing sugars were observed in the dwarf type. Tall type had more reducing sugars and titrable acidity and a greater amount of seed protein than the dwarf type [28].

**Table 1:** Characteristics of tall and dwarf types of *Grewia asiatica* [28].

Content (%)	Tall	Dwarf
Edible portion	91.30	90.79
Seed	8.70	9.21
Juice yield	67.50	65.90
Pomace	32.50	34.10
Moisture	76.80	74.83
Total sugars	5.73	7.95
Reducing sugars	1.24	0.99
Non-reducing sugars	4.49	6.96
Titrable acidity	1.48	1.12
Fruit protein	3.13	1.89
Seed protein	8.75	7.00
Pulp protein	1.40	7.00

## 3. Compositional and Phytochemical Studies

### 3.1. Compositional Studies

Fruits of *G. asiatica* are low in calories and fat, and high in vitamins, minerals, and fiber [29]. The detailed nutritional profile of fruit has been given in Table 2.

**Table 2:** Nutritional values of fruit [29].

Nutrients	Values/100 g g
Protein (g)	1.57
Total lipid (fat) (g)	<0.1
Carbohydrate (g)	21.1
Ash (g)	1.1
Fibre (g)	5.53
Calcium (mg)	136
Iron (mg)	1.08
Phosphorus (mg)	24.2
Potassium (mg)	372
Sodium (mg)	17.3
Vitamin B1 (mg)	0.02
Vitamin B3 (mg)	0.825
Vitamin C (mg)	4.385
Vitamin B2 (mg)	0.264
Vitamin A (g)	16.11

All these components are essential for healthy life. Vitamin C helps in wound healing and collagen formation and increases iron absorption from meal. Presence of sufficient amount of fibre helps to lower chances of obesity, cardiovascular disease, diabetes and certain cancers. In a recent study, various standardization parameters of fruits, microscopical and macroscopical characters, and physicochemical parameters like total ash (5.16%), water soluble ash (2.5%), acid insoluble ash (1%), sulphated ash (0.66%), loss on drying (8.3%), swelling index (0.1) and foaming index (less than 100) have been evaluated. Successive extraction and cold maceration values of fruits (%; w/w) in various solvents like petroleum ether (1.6 and 3), benzene (2.1 and 3.3), chloroform (2.6 and 4), ethyl acetate (3.7 and 4.3) and methanol (30.9 and 45.6) were observed, respectively. Phytochemical screening revealed the presence of alkaloids, carbohydrates, glycosides, proteins and amino acids, saponins, steroids, acids, mucilage, fixed oils and fats. Fruits were observed for their characters under visible and ultralviolet light after treating with different chemical reagents and pharmacognostic parameters variable on the basis of their geographical origin were determined [30]. Previous pharmacognostic studies of fruit indicated extractive values for petroleum ether, benzene, ethyl acetate, methanol and distilled water as 0.6, 1.3, 1.5, 34.5 and 12.5%, respectively. Total ash, acid insoluble ash and water soluble ash were 3.0, 1.4 and 1.1%, respectively. The behavior of the powdered leaves with different chemical reagents was noted. Fluorescence characteristics of the powdered leaves and extract were observed under UV (254 and 366 nm) and visible light [15].

The fruits are excellent for making juice and squash, which are regarded as very nutritious beverages by indigenous people. A refreshing summer drink prepared from fruits, called *phalsay ka sharbat* is available in food-stores and is believed as a cardiac tonic. The fruit juices may be fortified with other nutrients to further enhance its nutritional contribution to the diet. The juice has a low glycemic index and may be taken to manage diabetes, because carbohydrates in low glycemic index foods break down more slowly. Moreover, low glycemic index foods are believed to lessen the risk of coronary heart disease, and obesity. Nutritionally essential amino acids such as threonine and methionine are present in pulp and seeds, respectively, whereas phosphoserine, serine, and taurine are the dominant amino acids in juice. The pulp contains higher concentrations of phosphoserine as compared to other free amino acids, while the hydrolysed product contained aspartic acid, glycine, and tyrosine in large amount [31]. Pigments and total soluble solids have been obtained from pomace. The highest yield of pigments and total soluble solids were obtained by addition of water (75%) to pomace [32]. A ready-to-serve (RTS) beverage from the fruit juice was formulated and standardized. It contained 25% juice and a Brix-acid ratio of 45:1 [33]. A syrup was prepared from fruit juice, mixing the clear juice with an equal amount of sugar and preserved with sodium benzoate [34]. Amino acids were measured in the hydrolyzed and unhydrolyzed (free) pulp and seed and related to determination of the degree of adulteration in fruit juice. Threonine was found in pulp but was missing in seed extract, whereas methionine was only present in seeds, indicating that the presence of methionine in fruit juice would be the result of adulteration. Phosphoserine, serine, and taurine were the dominant amino acids in juice [35]. *G. asiatica* fruits were analyzed for six micronutrients (Co, Cr, Cu, Ni, Zn and Fe) on fresh weight (FW) and dry weight (DW) (Table 3) [36]. Iron was present in the highest concentration, while cobalt was present in the lowest amounts. Micronutrients play an important role in various physiological and metabolic processes of the human body.

**Table 3:** Mineral contents of fruit [36].

Mineral	mg/100 g FW *	µg/100 g DW **
Cobalt	0.99	33
Chromium	1.08	36
Copper	0.48	16
Nickel	2.61	87
Zinc	144	48
Iron	140.8	1695

* FW = Fresh weight (Fresh fruit); ** DW = Dry weight (After removal of moisture from fresh fruit).

Chemical composition of seeds indicated that they contain bright yellow oil (5%). Fatty acid composition of this oil indicated the presence of palmitic (8%), stearic (11%), oleic (13.5%) and linoleic acids (64.5%) while small amount of unsaponifiable matter (3%) was also detected [19].

### 3.2. Phytochemistry

#### 3.2.1. Preliminary Phytochemical Screening and Primary Metabolites

Phytochemical screening of the leaves revealed that their petroleum ether extract contains diterpenes, glycosides and fats; chloroform extract contains alkaloids and glycosides, while ethanolic extract contains triterpenoids, sterols, flavonoids, saponins and tannins [37]. Pharmacognostic evaluation of leaves reported total 5% of ash, consisting of water-soluble ash (2.5%) and acid-insoluble ash (2.1%). Other studies reported the extractive values of leaves in various solvents: petroleum ether (1.2%), benzene (1.2%), chloroform (1.6%), ethyl acetate (1.8%) and methanol (13.6%) [38]. Phytochemical screening of fruits indicated the presence of carbohydrate, tannins, phenolic compounds, flavonoids and vitamin-C in methanolic extract; flavonoids and fixed oil in petroleum ether extract; steroids in benzene extract; carbohydrate, tannins, flavonoids and phenolic compounds in ethyl acetate extract and carbohydrate, tannins, phenolic compounds and proteins in the aqueous extract [15]. Amino acids such as proline, glutaric acid, lysine and phenylalanine, and carbohydrates, like glucose, xylose, and arabinose were identified by paper chromatography in ethanol extract of fruit [39].

#### 3.2.2. Compounds Isolated and Secondary Metabolites

**Figure 2:**
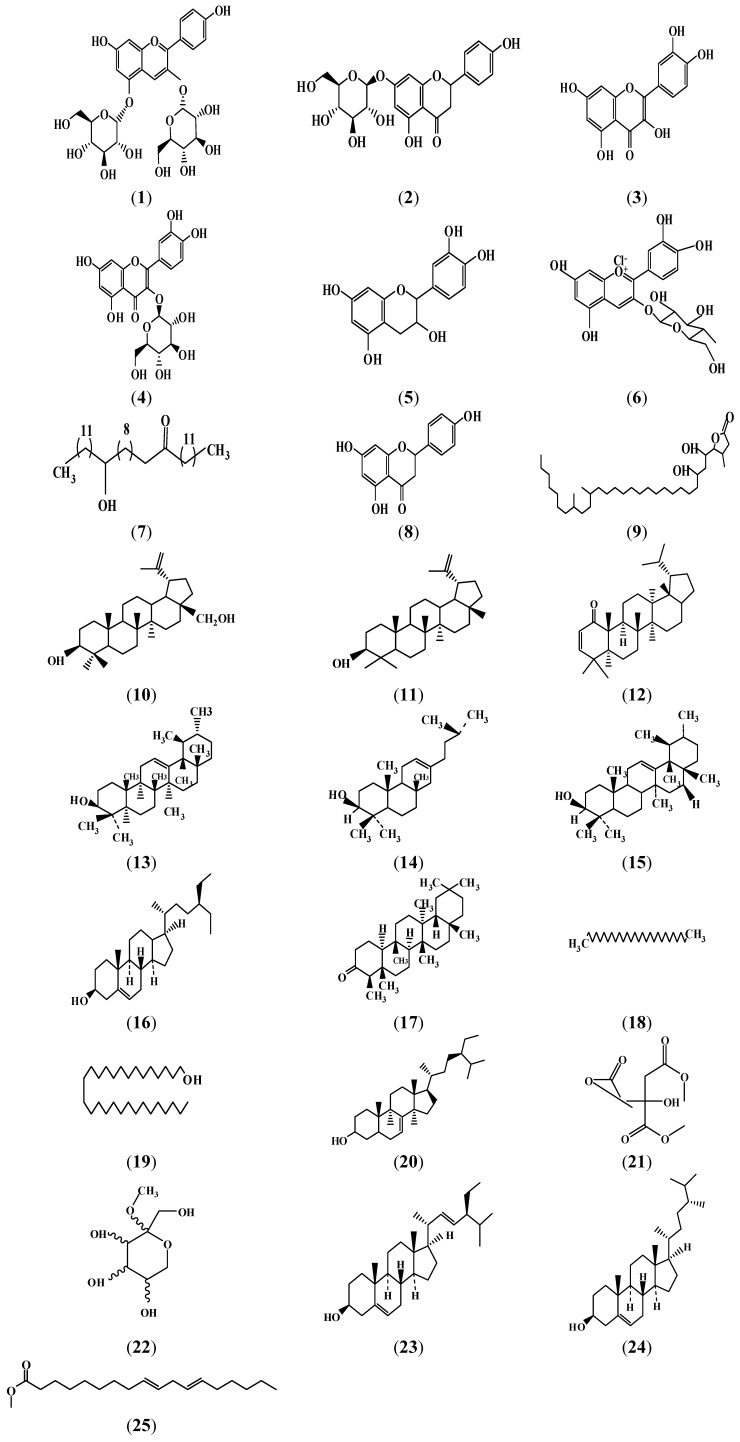
Compounds isolated from *G. asiatica*: Pelargonidin 3,5-diglucoside (**1**), Naringenin-7-*O*-β-D-glucoside (**2**), Quercetin (**3**), Quercetin 3-*O*-β-D-glucoside (**4**), Catechins (**5**), Cyanidin-3-glucoside (**6**), Grewinol (**7**), Naringenin (**8**), 3,21,24-trimethyl-5,7-dihydroxy-hentriacontanoic acid δ lactone (**9**), Betulin (**10**), Lupeol (**11**), lupenone (**12**), Friedelin (**13**), α-Amyrin (**14**), β-amyrin (**15**), β-sitosterol (**16**), Lanost-9(11)-en-12-one (**17**), Docosanol (**18**), Nonacosanol (**19**), Stigmast-7-en-3-ol (**20**), Citric acid trimethyl ester (**21**), α-methyl-l-sorboside (**22**), stigmasterol (**23**) Campesterol (**24**), 9,12-octadecadienoic acid methyl ester (**25**) [40,41,42,43,44,45].

Some secondary metabolites and isolated compounds present in *G. asiatica* are shown in Figure 2. Fruits of *G. asiatica* contain pelargonidin 3,5-diglucoside, naringenin-7-*O*-β-D-glucoside, quercetin, quercetin 3-*O*-β-D-glucoside, tannins, catechins, and cyanidin-3-glucoside [40]. Grewinol and its derivatives were isolated from the dried flowers [41]. Similarly β-sitosterol, quercetin, quercetin 3-*O*-β-D-glucoside, naringenin, naringenin 7-*O*-β-D-glucoside and a δ-lactone 3,21,24-trimethyl-5,7-dihydroxy-hentriacontanoic acid were isolated from flowers [37,42]. The stem bark contains betulin, lupeol, lupenone, and friedelin. β-amyrin and β-sitosterol were isolated from heartwood of *G. asiatica* [43]. Quercetin, kaempferol and a mixture of their glycosides were isolated from leaf extracts [44]. Citric acid trimethyl ester, α-methyl-l-sorboside , stigmasterol, campesterol and 9,12-octadecadienoic acid methyl ester were the main compounds identified in *G. asiatica* pomace extract [45].

## 4. Pharmacological Activities

### 4.1. Antioxidant Activity

*Grewia asiatica* has a high content of antioxidants like vitamin C, total phenolics, flavonoids, tannins and anthocyanins (Table 4). Much research has been conducted during last seven years on exploring the antioxidant effects of *G. asiatica*. Antioxidant activity of fresh and stored samples of seed, peel and pulp of fruit, extracted with five different solvents, was compared by using ABTS and modified DMPD decolorization assays (Table 5, Table 6) [46]. The results indicated that fruits and its various parts have higher antioxidant activity in fresh form as compared to frozen form. Trolox equivalent antioxidant capacity (TEAC) values showed that polyphenols in fresh *G. asiatica* samples have a potent *in vitro* antioxidant activity which may be related to its medicinal properties against diseases like diabetes mellitus and hepatitis. The highest values of antioxidant activity were obtained for peel followed by pulp and seeds.

**Table 4:** Total phenolic contents of various parts of fresh and frozen fruit (mg GAE/100 g) [46].

Solvent used	Seed	Peel	Pulp
70% Acetone	1020	5080	2060
Water	991	3990	2050
60% Methanol	920	2852	1261
Dichloromethane	880	1980	1000
Ethyl acetate	2060	1094	895

**Table 5:** ABTS antiradical activity (TEAC μmol/g) of various parts of fresh and frozen fruit [46].

Solvent used	Seed		Peel		Pulp	
	Fresh	Frozen	Fresh	Frozen	Fresh	Frozen
70% Acetone	19.6	8.7	107.2	56.8	60.9	29.5
Water	28.56	15.6	91.4	53.39	54.0	20.89
60% Methanol	49.0	19.3	87.8	33.13	56.1	25.8
Dichloromethane	25.3	11.5	69.4	24.90	29.6	15.8
Ethyl acetate	55.8	24	37.4	14.80	25.3	14.7

**Table 6:** DMPD antiradical activity (TEAC μ mol/g) of various parts of fresh and frozen fruit [46].

Solvents used	Seed		Peel		Pulp	
	Fresh	Frozen	Fresh	Frozen	Fresh	Frozen
70% Acetone	6.6	1.8	29.4	6.1	15.8	5.7
Water	9.8	1.3	24.0	3.6	11.0	5.0
60% Methanol	10.1	n.d.	18.6	n.d.	10.9	3.7
Dichloromethane	8.25	n.d.	17.7	3.0	9.4	n.d
Ethyl acetate	11.25	n.d.	10.5	n.d.	8.04	n.d

n.d = not detected.

Polyphenolics were isolated from crude methanol extracts of *G. asiatica* pulp and peel. The extracts were fractionated in two main fractions, anthocyanin fraction (Fraction II) and non-anthocyanin fraction (Fraction I) which was further divided into three groups: neutral fraction A, comprising flavanols and other polyphenolics (fraction Ib), neutral fraction B comprising flavonols (Fraction Ic), and acidic phenolics fraction (Fraction Ia). Table 7 reports total phenolic contents (TPC), total flavonoids contents (TFC) and total anthocyanin content of these fractions [47].

**Table 7:** Total phenolic, flavonoids and anthocyanin contents (mg/100 g) [47].

G. asiatica	TPC	TFC	TAC
Fraction Ia	67	n.d	n.d
Fraction Ib	288	178	72
Fraction Ic	222	165	n.d
Fraction II	151	100	n.d

n.d = not detected.

Extracts of leaves were screened for their *in vitro* antioxidant properties by assessing its potential to quench the two common radicals, DPPH and NO (Table 8). The results expressed as IC_50_ μg/mL were found to be comparable with standards such as an ascorbic acid and quercetin. The leaves were found to possess strong antioxidant activity [48].

**Table 8:** Antiradical activity (IC_50_ = μg/mL) of leaves [48].

Solvent used	DPPH	NO
Petroleum ether	249.60	22.12
Benzene	16.19	27.00
Ethyl acetate	26.17	47.38
Methanol	27.38	56.85
Methanol (50%)	56.40	72.75
Water	176.14	152.75

The antioxidant activity of a methanol extract of the fruit of *G. asiatica* was evaluated by various assays indicated that fruit possesses considerable antioxidant activities. Higher amounts of total flavonoid content (4.608 QE mg/g), total phenolic content (144.11 mg GAE/g) and total antocyanin contents (4.882 mg/kg) were observed, while the antiradical activity against DPPH (84.83%) and peroxide radical (37%) was observed. Values noted for TEAC (269.038 mMTE) and FRAP (4.14 GAE/g) were also comparatively greater than that of various other plant species [49].

The fruit pomace was assessed for total flavonoids, alkaloids, saponins and tannins and the values observed per dry matter were 12.42 ± 0.56 (CE mg/g), 1.56 ± 1.2 (g/100 g), 1.05 ± 0.96 (g/100 g) and 0.52 ± 1.25 (g/100), respectively [50]. These results indicate that even material considered as a waste has substantial amount of antioxidants.

An aqueous extract of fruit exhibited total phenol content (CE) and total flavonoid content (GAE) of 5.25 and 0.13, respectively. The results indicated comparatively higher contents as compared to other 21 extracts analyzed simultaneously [51]. It is well known that the antioxidant capacity, the total phenolic contents, the total flavonoid contents, the total tannin contents and the anthocyanin contents in plants depend on several factors such as different genotype, growing condition, agronomic practices employed, season, maturity, post-harvest storage and processing conditions and solvent used for extraction. These factors may explain the differences found among studies by different authors.

### 4.2. Radioprotective Effect

The increasing use of nuclear radiation for human welfare, despite its beneficial effects, has some undesirable side effects. There is dire need to check or minimize these side effects and for this reason scientists are seeking for new, safe, and cost-effective radioprotectors for persons who are in direct contact with these radiations. A research group based in India has done extensive work in this area and found encouraging results by using *G. asiatica* fruit extract as a natural radioprotective agent [18,23,52,53,54,55,56,57,58,59]. The effects of methanolic extract of fruit were evaluated in brains of Swiss albino mice for their radioprotective effects. The mice was divided in different groups: group I received no treatment, group II was orally supplemented, once daily, of the dose of 700 mg/Kg for fifteen consecutive days; group III (control) received distilled water orally equivalent to the extract for fifteen days, then was exposed to 5 Gy of gamma radiation, and group IV, to which the extract was administered orally for 15 consecutive days, once daily, and exposed to single dose of 5Gy of gamma radiation. Mice were sacrificed at different post irradiation intervals (1, 3, 7, 15 and 30 days). Brains were removed for the estimation of glutathione (GSH) and lipid peroxidation (LPO). Extract supplementation controlled the increase of LPO due to radiation, approximately by 5% at day 30 post irradiation, whereas radiation induced depleted levels of GSH could be raised by 14.57% 30 days after [52], thereby indicating that the extract may control the radiation induced disturbances. The radioprotective efficacy of the same extract against whole body gamma radiation was studied in Swiss albino mice. After drug toxicity testing, the oral administration of 700 mg/kg/day of extract for 15 consecutive days before exposure to 10 Gy of γ- radiation provided maximum protection, as evidenced by the highest number of survivors 30 days post irradiation. LD_50/30_ value of 6.21 for irradiation alone (control) and 9.53 for *Grewia asiatica* extract + irradiation group (experimental) was obtained, while the dose reduction factor calculated was 1.53. The mice of experimental group exhibited significant modulation of radiation-induced decreases of reduced glutathione (GSH) and radiation-induced increase in lipid peroxidation (LPO) in the whole brain and liver at 24 h after radiation exposure [53].

The radioprotective effect of a fruit pulp extract of *G. asiatica* in mice blood against radiation induced hematological and biochemical alterations was evaluated. Mice were divided into four groups, group I (normal) without any treatment, group II orally supplemented with extract once daily at the dose of 700 mg/kg for 15 days, group III (control) only irradiated and group IV (drug+IR) to which the extract was administered as to group II and then exposed to 5 Gy of gamma radiation. Mice were sacrificed at 24 and 72 h post irradiation. Radiation induced deficit in different blood constituents GSH, GSH-Px, sugar, and protein levels in serum were significantly increased, whereas radiation induced elevation of lipid peroxidation and cholesterol level was markedly decreased in extract pretreated animals than control group. The extract provided protection against radiation-induced alterations in blood of Swiss albino mice [54].

Radioprotective efficacy of a fruit extract was studied against radiation induced biochemical alterations in mice cerebrum. Mice were sacrificed at different intervals (1, 3, 7, 15 and 30 days) and cerebrum was tested for the estimation of glutathione (GSH), lipid peroxidation (LPO) and proteins. The extract showed a protection against the biochemical changes in mice cerebrum. Radiation induced increase in the levels of LPO was significantly reduced by extract post-treatment. Similarly, radiation-induced depletion in proteins was significantly controlled by extract administration [55].

In another study, the radioprotective effects of a fruit pulp extract on cerebrum of Swiss albino mice exposed to 5 Gy gamma radiation were investigated. Cerebra of mice was observed for various parameters after sacrificing in interval times of 1–30 days. Radiation-induced increase in the levels of lipid peroxidation of mice cerebrum was significantly reduced by extract pretreatment [56]. The radioprotective effects of the same extract were investigated in mice blood and liver, by the evaluation of glutathione (GSH) and lipid peroxidation. The results indicated that extract post-treatment protects liver and blood against radiation-induced damage, by inhibiting glutathione depletion and decreasing lipid peroxidation levels that attended normal levels by day 30 post-treatment. The magnitude of recovery from oxidative damage in terms of TBARS and GSH content was significantly higher (*p* < 0.001) in the irradiated + extract-treated group [57].

The radioprotective effects of a fruit extract were studied in Swiss albino mice divided into five groups: control (I); extract treated (700 mg/kg for 15 days) (II); irradiated (5 Gy) (III); extract + irradiated (IV) and irradiated extract treated (V). The irradiation of animals resulted in a significant elevation of lipid peroxidation in terms of thiobarbituric acid reactive substances (TBARS) content and depletion in glutathione (GSH) and protein levels, as compared to control group. The treatment of mice with extract before and after irradiation caused a significant depletion in TBARS content, followed by a significant elevation in GSH and protein concentration in the intestine and testis of mice at all post-irradiation autopsy intervals, in comparison to irradiated mice. Significant protection of DNA and RNA in testis was also noticed. The extract was found to have strong radical scavenging activity in DPPH and O^2^^−^ assays and also showed *in vitro* radioprotective activity in protein carbonyl assay, in a dose-dependent manner [18]. Biochemical, histopathological and behavioural changes after 5Gy whole body irradiation and its modulation by the same extract were studied in Swiss albino mice. Radiation induced changes in cerebellar lipid peroxidation (LPO), glutathione (GSH), protein, nucleic acids and histopathological changes were significantly (*p* < 0.001) addressed, especially at later intervals by extract administration pre- and post-irradiation. Radiation induced deficits in memory and learning were also significantly (*p* < 0.001) ameliorated. Results showed that the pre- and post-administration of *G. asiatica* extract has radioprotective potential as well as neuroprotective properties against radiation [23]. The radioprotective effect of the same extract was studied in mice testis by histopathological examination. Irradiation of animals led to significant decrease in testis weight, whereas the treated group showed significantly higher values in comparison to the irradiated group. The histopathological study showed that the group irradiated showed significantly lower spermatogonia “A”, spermatogonia “B”, spermatocytes and spermatid count. These counts were higher in extract pre- and post treated irradiated group, in comparison to the respective irradiated group, till last autopsy interval (30 days post irradiation). This can indicate that the extract has protective potential to the damaging effect of radiation to the testis [58].

The hepatoprotective effect of a fruit extract was studied in mice testis. The irradiation resulted in a significant decrease in DNA and RNA levels in comparison to controls. Administration of extract before and after irradiation caused a significant elevation in liver DNA and RNA levels. Photomicrography of liver showed that pre- and post- administration of extract provided protection against radiation [59].

### 4.3. Anticancer Activity

Aqueous extracts of leaves and fruits showed significant anticancer activity against liver cancer and breast cancer. The *in vitro* cytotoxic activity was determined by methylthiazolyl tetrazolium (MTT) assay using epidermal kidney (HEK-293), breast (MCF-7), cervical (HELA), lung (NCI-H522) and laryngeal (Hep-2) cancer cell lines. The fruit extract was found to be active on lung (IC_50_ = 59.03 µg/mL) and breast (IC_50_ = 58.65 µg/mL) cancer cell lines, while the leaf extract was active against breast (IC_50_ =50.37 µg/mL) and Hep-2 (IC_50_ = 61.23 µg/mL) cancer cell lines. The results suggest leaf and fruits extract as a potential agent for the management of human cancer [60].

The *in vitro* antitumoral and cytotoxic activities of a methanol extract of *G. asiatica* leaves has been assessed by MTT assay against four human cancer cell lines: acute myeloblastic leukemia (HL-60), chronic myelogenic leukemia (K-562), breast adenocarcinoma (MCF-7) and cervical epithelial carcinoma (Hela), with IC_50_ values of 53.70, 54.90, 199.5 and 177.8, 89.12, respectively. The intraperitoneal administration of 250 and 500 mg/kg of extract to male Swiss albino mice increased the life span of Ehrlich’s ascites carcinoma (EAC) tumor bearing mice by 41.22% and 61.06%, respectively. The same extract was found to be active in preventing the EAC development in mice in a dose dependent manner [61].

The *in vitro* cytotoxic activity (IC_50_) of pomace methanol extract evaluated against cervical epithelial carcinoma (HeLa), breast adenocarcinoma (MCF-7) and hepatocellular carcinoma cells (HepG-2) was >100, 68.91 and >250 µg/mL, respectively. These results suggested that *G. asiatica* pomace possessed promising anticancer activity that substantiated its ethno-medicinal use and may provide new molecules for treatment of these cancers [50].

### 4.4. Antimicrobial Activity

*G. asiatica* leaves possess antimicrobial potential and are therefore used to treat skin rashes and pustular eruptions [25]. Ethanolic extract of leaves showed antibacterial and antifungal activities. The extract showed potent results against eight bacterial strains; *Proteus mirabilis*, *Citrobacter* sp., *Pseudomonas aeruginosa*, *Escherichia coli*, *Salmonella typhi*, *Micrococcus luteus*, *Staphylococcus aureus*, and *Bacillus subtilis*. The extract showed moderate as well as significant activity against nine fungal strains namely *Aspergillus effusus*, *A. parasiticus*, *A. niger*, *Saccharomyces cerevisiae*, *Candida albicans*, *Yersinia aldovae*, *Fusarium solani*, *Macrophomina phaseolina*, and *Trichophyton rubrum* [8].

Polyphenolics were isolated from crude methanol extracts of *G. asiatica* pulp and peel and further fractionated into ethyl acetate fraction. This was further divided into three groups: neutral fraction A, comprising flavanols and other polyphenolics, neutral fraction B comprising flavonols, acidic phenolics fraction and anthocyanin fraction. These major fractions were analyzed for their antimicrobial effects. All fractions showed significant antibacterial activity, except the fraction containing anthocyanins. The most susceptible strain was *Staphylococcus* *aureus* amongst the Gram-positive, while amongst the Gram-negative bacterial strains, the most susceptible was *Salmonella typhi*. The most resistant Gram-positive bacteria was *Bacillus subtilis*, while most resistant Gram-negative strain was *E. coli*; both *Aspergillus* strains were substantially inhibited by all fractions. Fraction containing flavanols and other polyphenos was evaluated for its antifungal potential. No growth of *Trichophyton* *mentagrophytes* and *T. rubrum* was observed. Inhibition of *Aspergillus* strains by the fractions supports that the chemicals present in the fractions could be effective in the prevention of aflatoxin production in food products. Being the most active, phenolic acid fraction was also tested for its antifungal activity against six fungal pathogens, namely *Penicillium notatum*, *Aspergillus niger*, *A. flavus*, *Microsporum gypseum*, *Trichophyton mentagrophytes* and *T. rubrum*. The fraction substantially inhibited all the tested fungal species [47].

An ethanol extract of G. asiatica bark and fruit was tested against *Bacillus subtilis*, *Staphylococcus aureus*, *S. epidermidis* and *Streptococcus pneumoniae* and six Gram negative strains, *Escherichia coli*, *Proteus vulgaris*, *P. mirabilis*, *Salmonella typhi* para A, *S. typhi* para B and *Shigella dysenteriae*, resulting active against *S. aureus*, *E. coli* and *P. vulgaris* [62].

Different extracts of *G. asiatica* pomace were assayed against four Gram positive (*Bacillus subtilis*, *B. cereus*, *Staphylococcus aureus*, *Enterococcus faecalis*) and five Gram negative bacteria (*Escherichia coli*, *Listeria monocytogeneses*, *Salmonella typhimurium*, *Shigella flexneri* and *Pseudomonas aerugenosa*). Gram positive were more susceptible than Gram negative bacteria [45]. Gram-positive bacteria are usually more sensitive to crude extracts and bioactive constituents because of the specific structure of their cell walls.

### 4.5. Antiviral Activity

Sangita and coworkers [63] reported the antiviral activity of an extract of *G. asiatica* leaves against Urdbean Leaf Crinkle Virus (ULCV). The test plants previously sprayed with 500, 1,000, 1,500 and 2,000 μg/mL of *G. asiatica* extract were recorded as 58, 34, 38 and 48% of virus infection, respectively, in comparison to 90% infection of control. It was found the maximum inhibitory activity at 1,000 μg/mL and fairly good activity at concentrations of 1,500 and 2,000 μg/mL.

### 4.6. Antihyperglycemic and Antidiabetic Activity

Ethanolic extracts of fruit, stem bark and leaves of *G. asiatica* showed antihyperglycemic activity in alloxan-induced hyperglycemic rabbits. Oral administration in suspension and capsule at 200 mg/kg and 100 mg/kg, respectively of fruit, stem bark and leaves extract reduced serum glucose levels of rabbits. The results suggest that the fruit, stem bark and leaves of *G. asiatica* possess significant antihyperglycemic activity. Blood glucose, blood cholesterol and triglycerides levels were found to be significantly reduced by ground herbal drugs including *G. asiatica* (bark) in normal and alloxan induced diabetic rabbits [64]. Patil and coworkers [37] reported different extract of *G. asiatica* leaves for their hypoglycemic activity on alloxan induced diabetic wister rats. Ethanol extracts (200 mg/kg) showed more significant reduction in blood glucose level in alloxan induced diabetic Wister rats in comparison to control and the standard drug, glibenclamide.

Aqueous extracts of leaves were administered orally (250 mg/kg and 500 mg/kg) to normal rats and streptozotocin (STZ) (50 mg/kg) treated diabetic rats. Administration of extracts for 21 days significantly reduced blood glucose level in STZ induced diabetic rats. The plant extract was evaluated by oral glucose tolerance test model for its influence at different doses on blood glucose levels in normal rats fed with overload of glucose. Extracts significantly reduced the blood glucose level in a dose dependant manner. The results suggested that aqueous extracts significantly increased the glucose tolerance in normal rats [65]. Aqueous extracts of fresh fruits were studied for carbohydrate digesting enzymes (α-glucosidase and α-amylase) inhibitory properties. IC_50_ values (mg/mL) against α-amylase and α-glucosidase were 8.93 and 0.41, respectively, resulting in a moderate α-amylase and high α-glucosidase inhibitory activities as compared to other 21 extracts [51].

Fruit pomace was extracted with aqueous acetone (80:20), aqueous methanol (80:20) and a solvent mixture (ethanol/hexane/water, 80:10:10). All these three extracts were tested for their potential antidiabetic activity by α-amylase inhibition assay. IC_50_ (mg/mL) values observed are 45.7, 85.2 and 138.1, respectively [50]. Inhibition of α-amylase is believed as a strategy for diabetes and obesity management as it reduces blood sugar level.

### 4.7. Effect on Glycemic Index

In a recent study, Mesaik and coworkers [66] investigated the effects of *Grewia asiatica* fruit on glycemic index (GI) and phagocytosis in healthy non-diabetic human subjects. The results indicated that *Grewia asiatica* fruit has low GI value with modest hypoglycemic activity. The aqueous, methanol and butanol extracts of *Grewia asiatica* fruits were found to produce a stimulatory effect on ROS production; the chloroform, hexane and ethanol-acetate extracted exerted significant inhibitory effects. *Grewia asiatica* fruit has desirable effects on blood glucose metabolism manifested as low glycemic response and modulation of ROS production.

### 4.8. Anti-Hyperlipidemic Activity

Leaves were investigated for their anti-hyperlipidemic activity in induced-hyperlipidemic rats. The data suggested that extract had potent anti-hyperlipidemic effects. Fifty compounds were identified, of which six triterpenes, two sterols, one diterpene and four fatty alcohols were isolated. However, it has not been established which compound is responsible for the anti-hyperlipidemic effects [43].

### 4.9. Analgesic activity

The analgesic activity of an aqueous extract of fruit was evaluated by acetic acid induced writhing test and hot plate methods. Swiss albino mice were treated by various doses of the extract (100, 150, 200, 250 and 300 mg/kg). The extract, at doses from 100 mg to 250 mg/kg, exhibited significant inhibitory effects on acetic acid induced pain, while at dose of 300 mg/kg showed a good inhibitory effect, similar to aspirin. In hot plate method, the fruit extract showed significant inhibitory effect at dose of 100 mg/kg and greater inhibitory effects were observed at 300 mg/kg dose, while more potent inhibitory effects even greater than aspirin were noted at dose of 400 mg/kg [67].

### 4.10. Antipyretic Activity

Antipyretic effects of fruit extracts were observed in Swiss albino rats. Fever was induced by an intraperitonial injection of lipopolysaccharide (0.01 mg/mL) extracted from *E. coli*. Rectal temperature of each animal was recorded at 30, 60 and 90 min. An aqueous extract of fruit at doses from 300 to 500 mg/kg exhibited antipyretic activity greater than aspirin (100 mg/kg) within 30 min after administration [67].

### 4.11. Anti-Inflammatory Activity

A methanol extract of fruit was screened for its possible anti-inflammatory activity on carrageenan induced edema in rat paw at doses of 250 and 500 mg/kg, orally. The extract showed significant (*p* < 0.001 and *p* < 0.01) anti-inflammatory activity at both doses [68].

### 4.12. Antimalarial and Antiemetic Activities

Yaqeen and coworkers [69] reported the evaluation of the antiemetic activities of alcoholic extracts of fruits of *Grewia asiatica* in dog, whereas acute oral toxicity test was carried out in mice and rats. Oral dose of 200 mg/kg and 600 mg/kg of a crude alcoholic extract was found non-toxic in mice and rats. Oral dose of crude alcoholic extract (120 mg/kg body weight) caused antiemetic effect in dogs in 3 h and controlled emesis centrally induced by apomorphine (0.044 mg/kg body weight). This activity was comparable to standard commercial anti-emetic drugs like Maxolon (metoclopramide) and Largactil (chlorpromazine).

Zia-Ul-Haq and coworkers [25] reported the antimalarial and antiemetic activities of methanolic extract of leaves. The study indicated that *G. asiatica* leaves are a potential source of antimalarial and antiemetic drugs. The crude methanol extract showed antimalarial activity, (69% inhibition). Methanolic extract was administered to male chicks at 50 mg/kg and 100 mg/kg dose levels and percent inhibition of emetic action was 39.14% and 59.69%, respectively.

### 4.13. Antiplatelet Activity

There is a great interest in exploring the anti-platelet activity of medicinal plant extracts because these are inexpensive and easily available from indigenous resources. Zia-Ul-Haq and co-workers [26] reported the anti-platelet activity of a crude methanol extract of *G. asiatica* L. leaves. The extract exhibited a potent platelet aggregation inhibition activity, in a dose-dependent manner at a concentration range of 1–10 mg/mL, suggesting that this extract can be considered as treatment for prevention of cardiovascular or inflammatory diseases.

### 4.14. Other Activities

Methanolic extract of leaves possessed nematicidal (against *Helicotylenchus indicus*), insecticidal (against *Tribolium castaneum*, *Callosbruchus analis* and *Rhyzopertha dominica*), phytotoxic (against *Lemna minor*), cytotoxic (against *Artemia salina*) and larvicidal (against *Haemonchus contortus*) activities [70].

## 5. Adsorption Studies

Water contamination by dyes and metal ions, due to increasing industrial activities, is becoming a serious environmental concern. Lead, due to its possible toxic effects and non-biodegradable nature, is recognized as a longstanding water contaminant. Due to Pb^2+^’s ability to react with phosphate ions of enzymes and mercapto groups, it inhibits the biosynthesis of haeme units, affects membrane permeability of liver, kidney and brain cells leading to their reduced functions or complete breakdown. Congo red dye is recognized as a skin, eye and gastrointestinal irritant. On decomposition, it produces carcinogenic amines which are harmful for life. Due to their potential hazardous environmental effects, toxicity to humans and to meet regulatory discharge standards, it is essential to remove lead from wastewater prior to discharge into fresh water bodies. Adsorption is a very effective waste water treatment technique due to its simplicity and ease of operation. *G. asiatica* leaves and seeds exhibited excellent adsorption potential in batch-wise adsorption experiments and may be used as effective, low cost and green alternative biosorbent material for lead and congo red dye removal from aqueous solution [71]. In a comparative study *G. asiatica* leaves exhibited better adsorption potential than *Raphanus sativus* peels for removing congo red dye from water and its efficiency of adsorption is comparable with charcoal [72].

## 6. Conclusions

*Grewia asiatica* is a food plant and can also be used as a herbal medicine for the treatment of various diseases such as cancer, ageing, fever, rheumatism and diabetes. This plant can also be used as an antioxidant and radioprotective agent. Its fruits possess a variable extent of antioxidant activity besides providing essential nutrients. Detailed investigation of this fruit should be carried out to utilize it in the most appropriate way. Studies mostly addressed the basic chemical and pharmacological characteristics of phalsa fruit. Detailed studies are needed for identifying, quantifying and deciphering bioactive constituents responsible for tagged activities. There is need to develop new varieties with big fruits having improved quality, sweetness and flavor, greater yield, pest resistant and adaptable to grow in colder regions.
